# Supplementary material for: Combined Transcriptome and Metabolome Analysis Reveals Adaptive Defense Responses to DON Induction in Potato
Source: Int J Mol Sci. 2023 Apr 29;24(9):8054. doi: 10.3390/ijms24098054 (PMC10179060; doi:10.3390/ijms24098054)
Supplement: Supplementary file 1 [file ijms-24-08054-s001.zip › Supplement Figures and Table S1-S5.pdf]

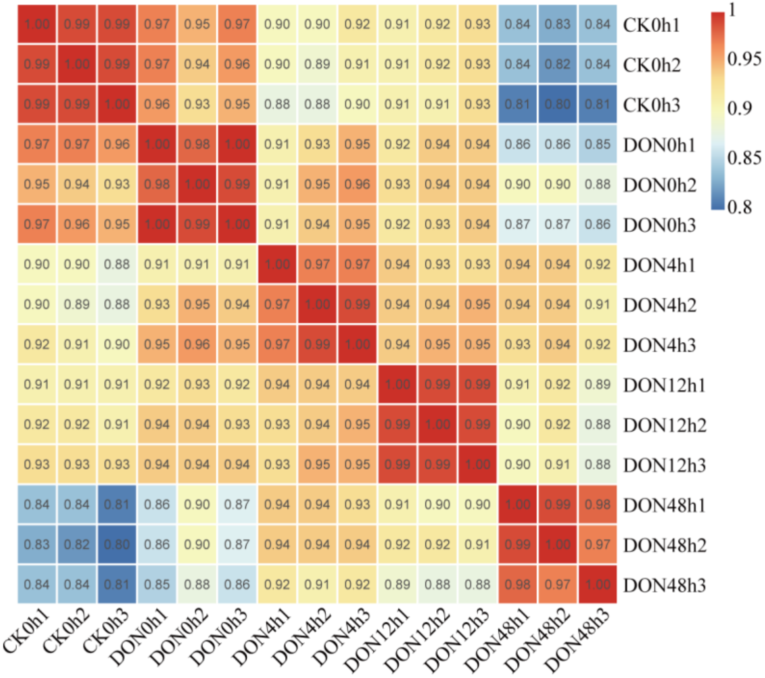

Figure S1. Correlation heatmaps of metabolomic samples.

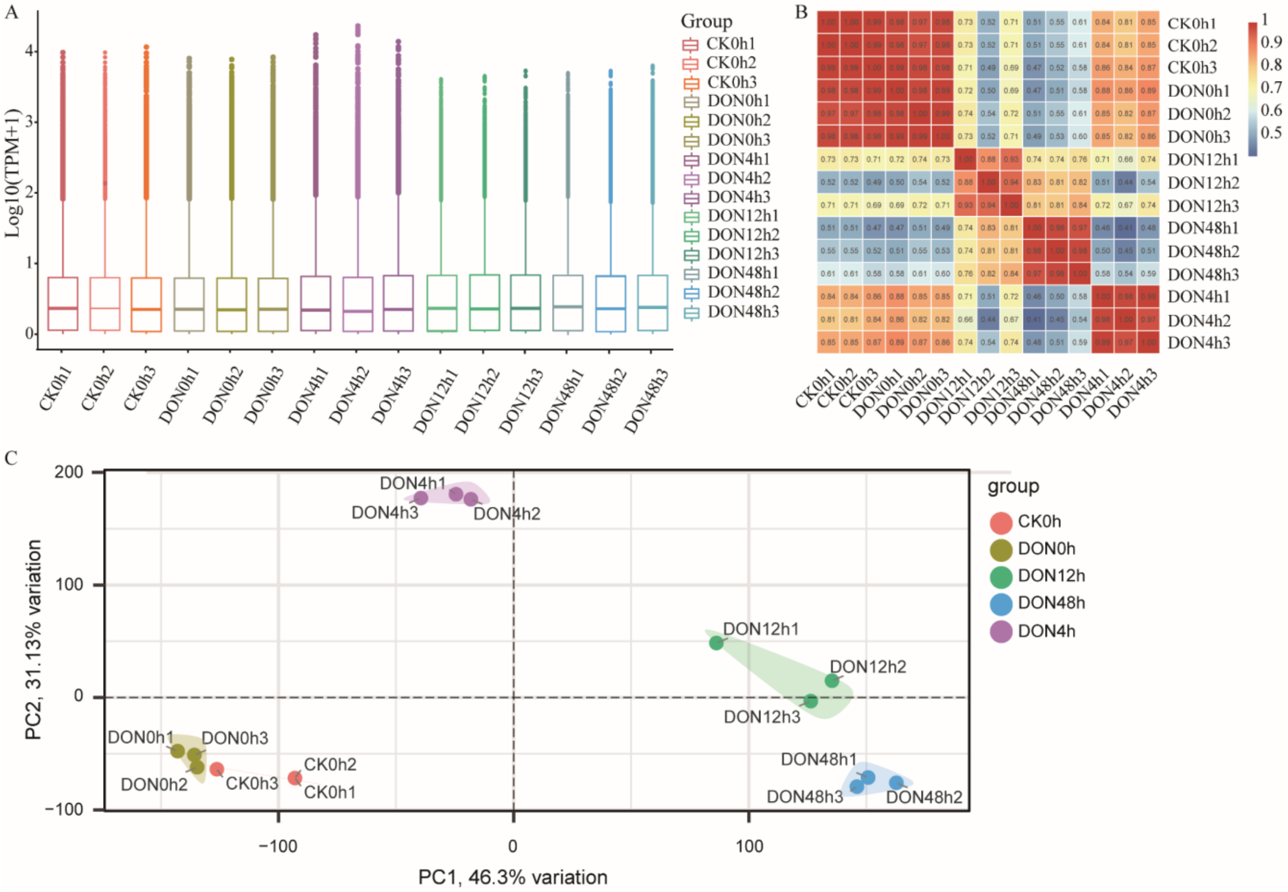

Figure S2. Gene expression and correlation analysis. (A) Single gene expression profiles of each treated sample. (B) Sample correlation heatmaps. (C) Principal component analysis.

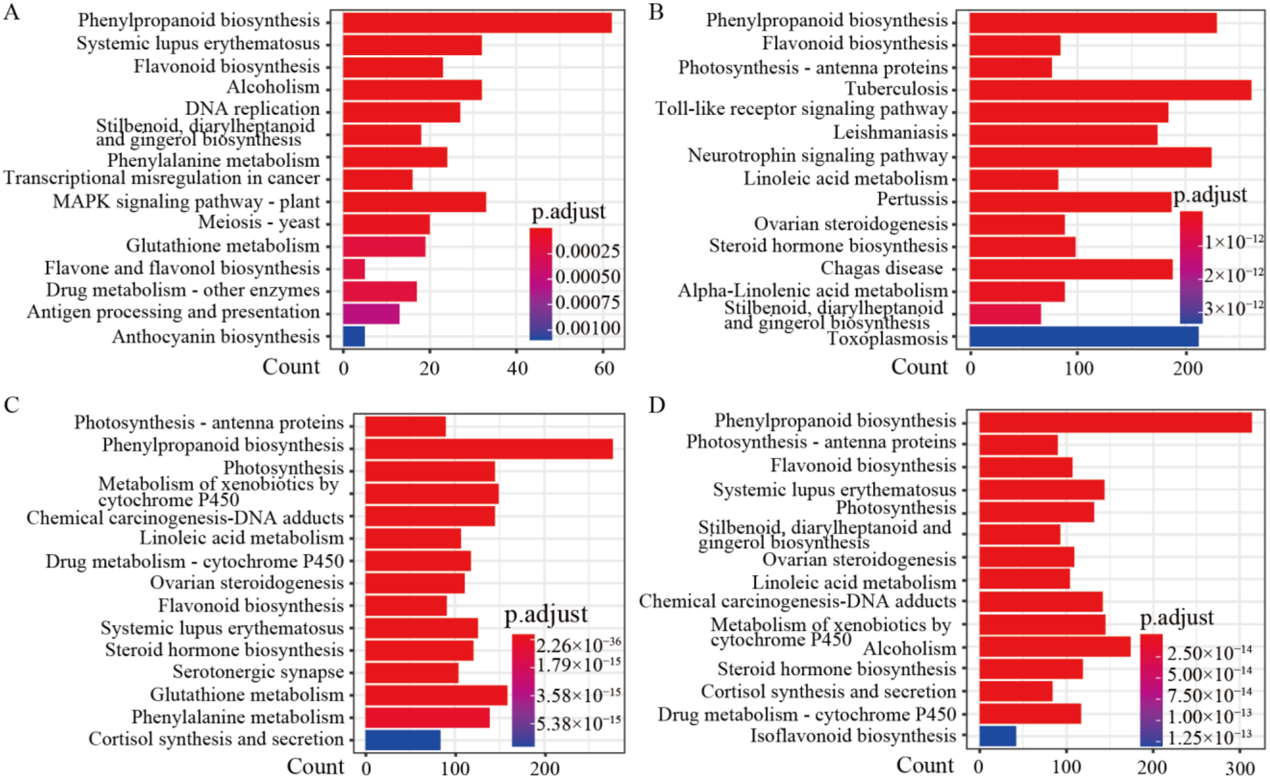

Figure S3. KEGG pathway enrichment map of DEGs under DON stress. (A) KEGG pathway enrichment map of DEGs in the CK0h vs. DON0h group. (B) KEGG pathway enrichment map of DEGs in the CK0h vs. DON4h group. (C) KEGG pathway enrichment map of DEGs in the CK0h vs. DON12h group. (D) KEGG pathway enrichment map of DEGs in the CK0h vs. DON48h group.

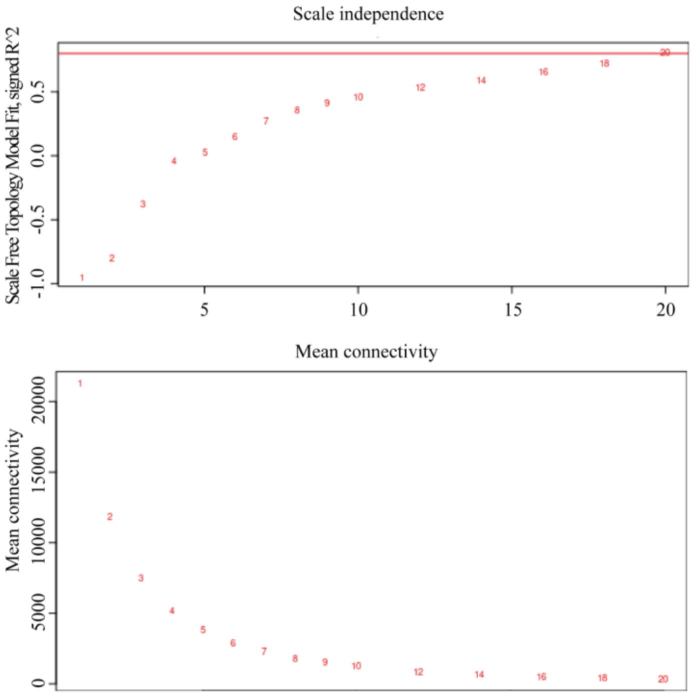

Figure S4. The choice of power. As close as possible to a scale-free network, preserving connectivity as much as possible.

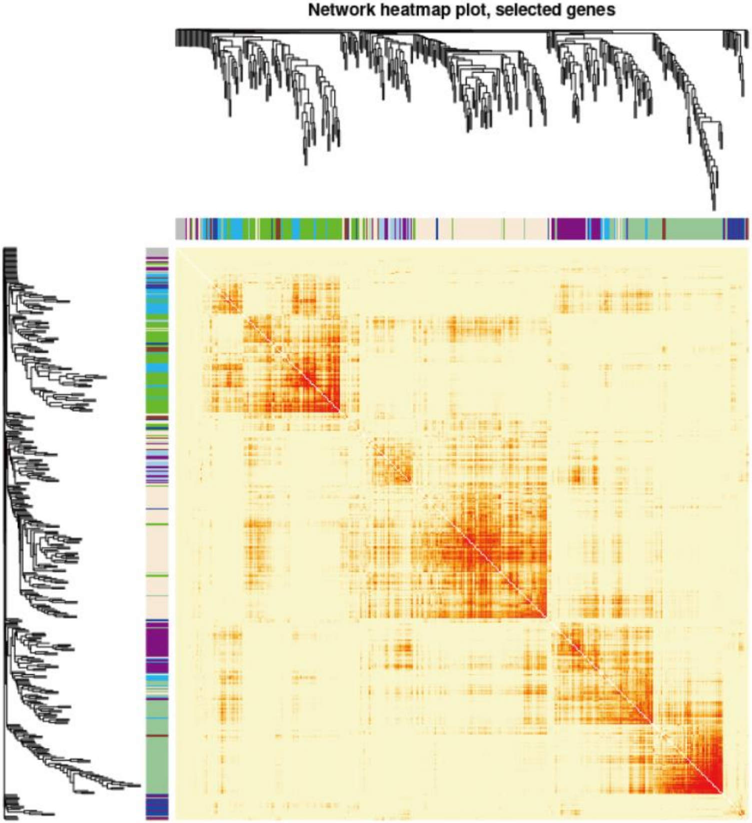

Figure S5. Gene co-expression network, make use of interaction patterns among genes.

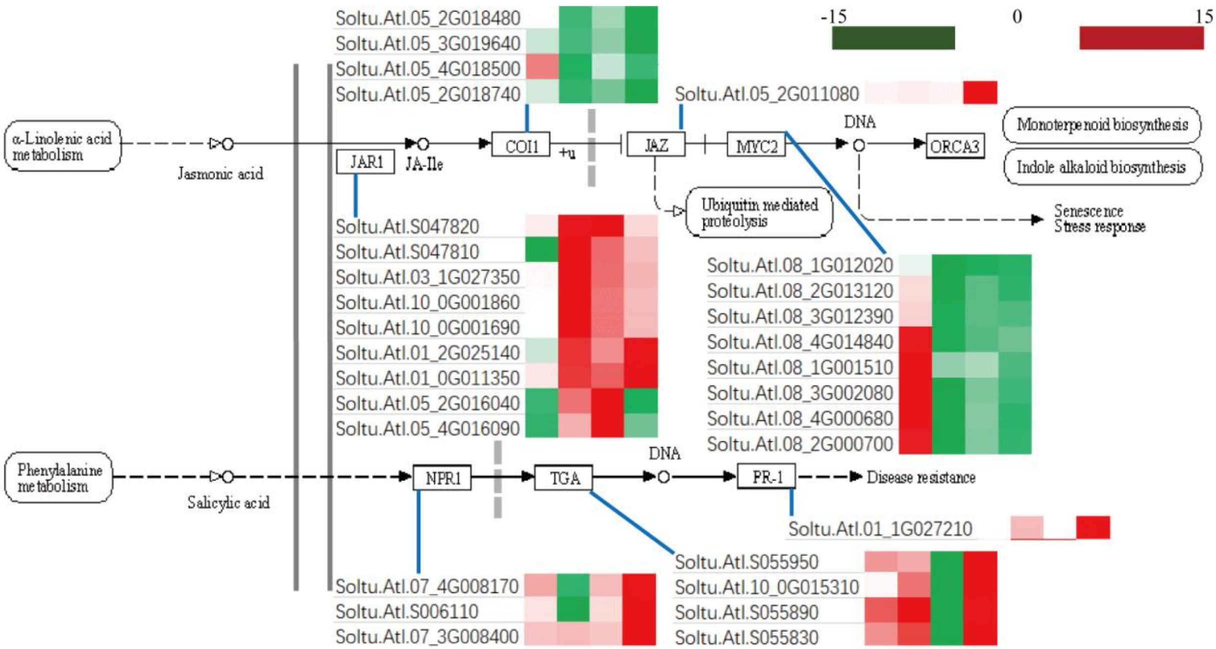

Figure S6. SA and JA signaling pathway maps. The heat map was drawn based on the TPM values in the transcriptome dataset. The columns and rows in the heat map represent samples and genes, respectively.

Table S1. Statistics of raw sequencing data components

Sample	Clean Reads	Adapter related	Containing too many N	Low Quality
CK0h1	91.98%	7.90%	0.12%	0%
CK0h2	92.15%	7.74%	0.12%	0%
CK0h3	91.60%	8.27%	0.13%	0%
DON0h1	90.94%	8.94%	0.12%	0%
DON0h2	92.21%	7.67%	0.12%	0%
DON0h3	92.02%	7.85%	0.13%	0%
DON4h1	90.45%	9.45%	0.10%	0%
DON4h2	91.43%	8.47%	0.10%	0%
DON4h3	86.82%	13.08%	0.10%	0%
DON12h1	90.77%	9.14%	0.09%	0%
DON12h2	91.29%	8.62%	0.09%	0%
DON12h3	90.57%	9.33%	0.10%	0%
DON48h1	90.57%	9.30%	0.13%	0%
DON48h2	89.93%	9.94%	0.12%	0%
DON48h3	89.50%	10.39%	0.12%	0%

Table S2. Data quality control status table

Sample Name	% Dups	% GC	Read Length	% Failed	M Seqs
CK0h1_1	53.10%	42%	150 bp	20%	27.5
CK0h1_2	53.00%	42%	150 bp	20%	27.5
CK0h2_1	52.00%	42%	150 bp	20%	26.3
CK0h2_2	51.80%	42%	150 bp	20%	26.3
CK0h3_1	53.70%	42%	150 bp	20%	23.9
CK0h3_2	54.60%	42%	150 bp	20%	23.9
DON0h1_1	50.90%	42%	150 bp	20%	24
DON0h1_2	51.00%	42%	150 bp	20%	24
DON0h2_1	53.20%	42%	150 bp	20%	25.8
DON0h2_2	53.60%	42%	150 bp	20%	25.8
DON0h3_1	52.00%	42%	150 bp	20%	26.5
DON0h3_2	53.00%	42%	150 bp	20%	26.5
DON12h1_1	53.30%	42%	150 bp	20%	25.9
DON12h1_2	51.00%	42%	150 bp	20%	25.9
DON12h2_1	54.20%	42%	150 bp	20%	25.8
DON12h2_2	52.20%	43%	150 bp	20%	25.8
DON12h3_1	54.30%	42%	150 bp	20%	26
DON12h3_2	52.80%	43%	150 bp	20%	26
DON48h1_1	50.30%	42%	150 bp	20%	26.4
DON48h1_2	51.00%	43%	150 bp	20%	26.4
DON48h2_1	50.50%	42%	150 bp	20%	24.6
DON48h2_2	51.00%	42%	150 bp	20%	24.6
DON48h3_1	51.60%	42%	150 bp	20%	27
DON48h3_2	51.20%	42%	150 bp	20%	27
DON4h1_1	56.40%	42%	150 bp	20%	25.9
DON4h1_2	54.80%	42%	150 bp	20%	25.9
DON4h2_1	57.70%	42%	150 bp	20%	25.3
DON4h2_2	56.30%	42%	150 bp	20%	25.3
DON4h3_1	57.40%	42%	150 bp	20%	28.6
DON4h3_2	55.40%	42%	150 bp	20%	28.6

Note: % Dups, Proportion of duplicate reads. M Seqs, Total sequencing volume, unit is millions.

Table S3. Summary of RNA-seq reads mapping

Sample	All pairs	Unique mapped	Unique mapped ratio	Multi mapped	Multi mapped ratio	All mapping pairs ratio
CK0h1	27,469,791	14,454,014	52.62%	11,704,669	42.61%	97.85%
CK0h2	26,316,511	13,764,641	52.30%	11,209,303	42.59%	97.62%
CK0h3	23,923,378	12,518,453	52.33%	10,273,710	42.94%	97.74%
DON0h1	23,954,662	12,497,163	52.17%	10,235,014	42.73%	97.52%
DON0h2	25,801,425	13,580,725	52.64%	11,033,006	42.76%	97.89%
DON0h3	26,498,602	13,919,720	52.53%	11,297,248	42.63%	97.70%
DON4h1	25,862,393	13,628,967	52.70%	10,649,487	41.18%	97.09%
DON4h2	25,259,003	13,078,486	51.78%	10,661,612	42.21%	97.23%
DON4h3	28,588,723	14,934,597	52.24%	11,846,167	41.44%	96.96%
DON12h1	25,910,393	13,614,021	52.54%	10,690,489	41.26%	97.11%
DON12h2	25,835,893	13,644,218	52.81%	10,584,339	40.97%	97.11%
DON12h3	26,035,310	13,621,916	52.32%	10,881,689	41.80%	97.28%
DON48h1	26,430,156	13,949,331	52.78%	11,069,088	41.88%	97.48%
DON48h2	24,640,904	12,837,420	52.10%	10,064,911	40.85%	95.85%
DON48h3	27,024,566	14,266,772	52.79%	11,198,489	41.44%	97.27%

Table S4. Hub genes information

Gene ID	Gene name	Abbreviations	Function	Cover Arabidopsis gene
Soltu.Atl.01_1G000250	TREHALOSE -6-PHOSPHATASE SYNTHASE S6	ATTPS6	Encodes an enzyme putatively involved in trehalose biosynthesis.	AT1G68020
Soltu.Atl.01_1G002310	ATP-BINDING CASSETTE G22	ABCG22	Encodes ABCG22, an ABC transporter gene.	AT5G06530
Soltu.Atl.08_2G012430	POLYPEPTIDE 36	CYP71B36	putative cytochrome P450.	AT3G26320
Soltu.Atl.01_0G011240	-	-	Galactose mutarotase-like superfamily protein.	AT3G61610
Soltu.Atl.05_3G017570	-	-	Tropomyosin.	AT1G33500
Soltu.Atl.01_4G000260	TREHALOSE -6-PHOSPHATASE SYNTHASE S6	ATTPS6	Encodes an enzyme putatively involved in trehalose biosynthesis.	AT1G68020
Soltu.Atl.02_4G007260	ATSR1 INTERACTION PROTEIN 1	SR1IP1	Encodes a phototropin-interacting NRL protein	AT5G67385
Soltu.Atl.06_4G022030	-	-	Nuclear pore localization protein NPL4.	AT2G47970
Soltu.Atl.08_3G005920	OXIDATION-RELATED ZINC FINGER 1	ATC3H20	Encodes Oxidation-related Zinc Finger 1 (OZF1).	AT2G19810
Soltu.Atl.05_3G007220	POLYGALACTURONASE 2	PG2,	Polygalacturonase involved in cell wall modification.	AT1G70370
Soltu.Atl.06_0G001780	MYB DOMAIN PROTEIN 48	ATMYB48	Encodes transcription factor MYB48.	AT3G46130
Soltu.Atl.08_4G007740	OXIDATION-RELATED ZINC FINGER 1	ATC3H20	Encodes Oxidation-related Zinc Finger 1 (OZF1).	AT2G19810
Soltu.Atl.04_1G008290	CYTOKININ OXIDASE/DEHYDROGENASE 1	ATCKX1	catalyzes the degradation of cytokinins.	AT2G41510
Soltu.Atl.01_4G001730	GLYCOSYLPHOSPHATIDYLINOSITOL-ANCHORED LIPID PROTEIN TRANSFER 14	LTPG14	Bifunctional inhibitor/lipid-transfer protein/seed storage 2S albumin superfamily protein.	AT2G44300
Soltu.Atl.S116020	ERYTHRONATE-4PHOSPHATE DEHYDROGENASE	E-4PDHASE	Tail-anchored (TA) OEP membrane protein.	AT1G19400
Soltu.Atl.05_2G010550	-	-	hypothetical protein.	AT4G26450
Soltu.Atl.05_4G002760	NRT1/ PTR FAMILY 3.1	ATNPF3.1	a membrane localized GA transporter.	AT1G68570
Soltu.Atl.07_1G016340	-	-	Rab3 GTPase-activating protein non-catalytic subunit.	AT3G14910
Soltu.Atl.02_3G026680	PSAA	-	Encodes psaA protein comprising the reaction center for photosystem I along with psaB protein.	ATCG00350
Soltu.Atl.01_2G035810	N-ACETYLSEROTONIN O-METHYLTRANSFERASE	ASMT	Encodes a cytosolic N-acetylserotonin O-methyltransferase .	AT4G35160
Soltu.Atl.03_2G020060	HIPL2 PROTEIN PRECURSOR	HIPL2	hipl2 protein precursor.	AT5G62630
Soltu.Atl.01_2G021120	VHA-D	-	Member of V-ATPase family.	AT3G58730
Soltu.Atl.09_4G021820	CYTOCHROME P450,	CYP76C2	member of CYP76C	AT2G45570
Soltu.Atl.S086480	VEGETATIVE STORAGE PROTEIN 3	VSP3	secreted acid phosphatase	AT4G29260
Soltu.Atl.02_3G027200	ABERRANT POLLEN DEVELOPMENT 2	APD2	RING/U-box superfamily protein.	AT5G01450
Soltu.Atl.01_2G032260	CALMODULIN 1	ACAM-1	encodes a calmodulin,Gene expression is rapidly induced upon a variety of abiotic stimuli.	AT5G37780

Soltu.Atl.04_2G018430	CYTOCHROME P450	CYP76C2	member of CYP76C	AT2G45570
Soltu.Atl.01_0G016100	PICLORAM RESISTANT30	PIC30	Major facilitator superfamily transmembrane transporter responsible for the uptake of picolinate herbicides	AT2G39210
Soltu.Atl.04_0G007890	MDIS1-INTERACTING RECEPTOR LIKE KINASE2	MIK2	MIK1 encodes a receptor kinase that forms a complex with MDIS1/MIK2 and binds LURE1, the female pollen guidance chemi-attractant. MIK1 phosphorylates MDIS1 and is autophosphorylated.	AT4G08850
Soltu.Atl.09_4G014460	PEROXIDASE 57	PER57	Peroxidase superfamily protein, overexpression increases ROS	AT5G17820
Soltu.Atl.03_3G002080	RECEPTOR KINASE 3	RK3	encodes a putative receptor-like serine/threonine protein kinases .	AT4G21380
Soltu.Atl.03_1G026210	ARIA-INTERACTING DOUBLE AP2 DOMAIN PROTEIN	ADAP	Encodes ADAP,ADAP is a positive regulator of the ABA response and is also involved in regulating seedling growth	AT1G16060
Soltu.Atl.01_3G032890	ATC3H50	-	FMN-linked oxidoreductases superfamily protein.	AT4G38890
Soltu.Atl.02_2G027420	SKU5 SIMILAR 17	SKS17	SKU5 similar 17	AT5G66920
Soltu.Atl.04_1G010150	CYCLIN-DEPENDENT KINASE G2	CDKG2	Cyclin dependent kinase. Regulates stress responses and FLOWERING LOCUS M mRNA splicing.	AT1G67580
Soltu.Atl.05_3G021100	ECERIFERUM 10	CER10	Enoyl-CoA reductase	AT3G55360
Soltu.Atl.03_2G015140	CAFFEATE O-METHYLTRANSFERASE 1	COMT1	A caffeic acid/5-hydroxyferulic acid O-methyltransferase.	AT5G54160
Soltu.Atl.09_4G020740	ACYL ACTIVATING ENZYME 16	AAE16	AMP-dependent synthetase and ligase family protein involved in fatty acid biosynthesis.	AT3G23790
Soltu.Atl.01_0G015940	-	-	Alpha-helical ferredoxin.	AT1G79010
Soltu.Atl.02_1G009850	LOB DOMAIN-CONTAINING PROTEIN 4	LBD4	LOB domain-containing protein 4.	AT1G31320
Soltu.Atl.02_4G018830	-	-	Translation protein SH3-like family protein.	AT3G49910
Soltu.Atl.04_2G020280	RDS2	-	HSP20-like chaperones superfamily protein.	AT1G76440
Soltu.Atl.02_1G027570	-	-	NADH dehydrogenase ubiquinone complex I, assembly factor-like protein (DUF185).	AT3G28700
Soltu.Atl.S120650	RBFA DOMAIN-CONTAINING PROTEIN 1	RBF1	ribosome-binding factor A family protein.	AT4G34730
Soltu.Atl.01_1G024450	RIBOSOMAL PROTEIN L18	RPL18	Encodes cytoplasmic ribosomal protein L18.	AT3G05590
Soltu.Atl.04_4G014150	ARABIDOPSIS THALIANA V-PPASE 3	ATAVP3	Encodes a H(+)-translocating (pyrophosphate-energized) inorganic pyrophosphatase (H(+)-PPase; EC 3.6.1.1) located in the vacuolar membrane.	AT1G15690
Soltu.Atl.S135260	PROTEIN TYROSINE PHOSPHATASE LOCALIZED TO MITOCHONDRION 1	PTPMT1	Encodes a phosphatidylglycerophosphate (PGP) phosphatase.	AT2G35680
Soltu.Atl.06_3G014480	DOM1	-	Encodes a protein DOMINO1 that belongs to a plant-specific gene family .	AT5G62440
Soltu.Atl.09_3G014990	MCCA	-	MCCA is the biotinylated subunit of the dimer MCCase, which is involved in leucine degradation.	AT1G03090

Soltu.Atl.S037690	-	-	Transducin/WD40 repeat-like superfamily protein.	AT3G15610
Soltu.Atl.08_4G006420	-	-	Ribosomal protein S3 family protein.	AT5G35530
Soltu.Atl.02_2G029400	-	-	Proteasome component (PCI) domain protein.	AT5G15610
Soltu.Atl.05_0G001050	FERREDOXIN-NADP(+) OXIDOREDUCTASE - LIKE	FNRL	oxidoreductase located in chloroplast	AT1G15140
Soltu.Atl.S146330	HYPOTHETICAL PROTEIN 22	HP22	PRAT protein family.	AT5G55510
Soltu.Atl.04_4G003260	-	-	Ribosomal protein L23/L15e family protein.	AT4G16720
Soltu.Atl.01_4G024730	RIBOSOMAL PROTEIN L18	RPL18	Encodes cytoplasmic ribosomal protein L18.	AT3G05590
Soltu.Atl.01_2G029130	RIBOSOMAL PROTEIN L18, RPL18	-	Encodes cytoplasmic ribosomal protein L18.	AT3G05590
Soltu.Atl.06_1G001600	RPL14B	-	Cytoplasmic ribosomal protein.	AT4G27090
Soltu.Atl.04_1G004040	-	-	Ribosomal protein L23/L15e family protein.	AT4G16720
Soltu.Atl.04_3G003300	-	-	Ribosomal protein L23/L15e family protein.	AT4G16720

Table S5. Primer sequences of qRT-PCR related genes

Primer Name	Forward Primer Sequences	Reversed Primer Sequences
β-action	GCTTCCCGATGGTCAAGTCA	GGATTCCAGCTGCTTCCATTC
ABCG22	GGTGGCAATCTGGTGGTGAT	TCCTTGTTCAGCATTGCCCT
CYP71B36	ACTGGAAACAGATGCGCAAA	ACAACATTTGGCGTAGAGCG
SR1IP1	TTGAAGGCACATCCAGCTCT	GGGAGCCTCTCATTCTGAGC
C3H20	GGGATGGTGAGAGGTATGCG	ATCGAACACCGACACTCCAC
PG2	CGGTAGAGATTCACCCCGTG	CCGTAGATCGGGGACATTGG
CKX1	GAGCACCAGTGATGCGTTTC	CCGGCATTCGACAAAGTACC
CYP76C2	CACCCACCAGCTCCATTCTT	GAATCACGACCAATTGCCCA
VSP3	TGCTTGGCAAACAATACCGC	ATGCAACATCAGAACGAGCG
APD2	ACAGGCTGCGTTAGAGTACG	AAGACGCACTTGCTTTCGAT
ADAP	ATACCGTGGACTAAACGCCG	CAGCCGGAGCTTTGTTGTTG
COMT	ATGCACCGGTAATGCTCGAT	GAGCAAAAGTGGGGCAACAG
RBF1	GTGAGCTAGGGAGGCGAATG	TCCATCATTGGCAGATCCCG
PTPMT1	ACGAAAAGGGCTTTGGTTGG	CCTAGAGCCTTCAAGCGAGG
WD40	GGGATGCATTGACTGGGGAT	GAGGTGCATCAGGTCGGTTT
L15e	TGGTCTACCGTGTTCGTGTG	ATTTCCTACCAGCACGCTCC
